# Neuroenhancement and the strength model of self-control

**DOI:** 10.3389/fpsyg.2015.01425

**Published:** 2015-09-24

**Authors:** Chris Englert, Wanja Wolff

**Affiliations:** ^1^Department of Educational Psychology, Institute of Educational Science, University of Bern, Bern, Switzerland; ^2^Department of Sport Psychology, University of Potsdam, Potsdam, Germany

**Keywords:** ego depletion, neuroenhancement, self-control, self-regulation

## Abstract

Neuroenhancement (NE), the use of substances as a means to enhance performance, has garnered considerable scientific attention of late. While ethical and epidemiological publications on the topic accumulate, there is a lack of theory-driven psychological research that aims at understanding psychological drivers of NE. In this perspective article we argue that self-control strength offers a promising theory-based approach to further understand and investigate NE behavior. Using the strength model of self-control, we derive two theory-driven perspectives on NE-self-control research. First, we propose that individual differences in state/trait self-control strength differentially affect NE behavior based on one’s individual experience of NE use. Building upon this, we outline promising research questions that (will) further elucidate our understanding of NE based on the strength model’s propositions. Second, we discuss evidence indicating that popular NE substances (like Methylphenidate) may counteract imminent losses of self-control strength. We outline how further research on NE’s effects on the ego-depletion effect may further broaden our understanding of the strength model of self-control.

## Introduction

A survey recently published in *Nature* revealed that one out of five respondents admitted having previously used substances as a means to enhance cognitive performance (Maher, 2008). The results of this survey fueled considerable research activity in the field of this so called *Neuroenhancement* (NE). We understand NE as a behavior that occurs within a defined means-end relation. This means a substance is being used as a means to enhance cognitive performance (Wolff and Brand, 2013; Wolff et al., 2014). *Drug Instrumentalization Theory* (DI-Theory) proposes that the means-end relationship that underlies such non-addictive drug use can be understood as a two-step process: “(1) the seeking and consumption of a psychoactive drug in order to change the present mental state into a previously learned mental state, which then allows for, (2) better performance of other, previously established behaviors and better goal achievement” (Mueller and Schumann, 2011). Understanding NE from the perspective of DI-Theory, an individual uses a substance with the aim of changing his or her current mental state (e.g., being tired and not concentrated) into a more desirable state (e.g., being alert and able to focus), which then allows for better performance. From a psychological perspective it is not important if the chosen substance is actually effective in enhancing performance. The assumed functionality attributed to a substance is seen as the driving force behind NE behavior (Wolff and Brand, 2013; Maier and Schaub, 2015). NE has therefore been defined as a healthy individuals’ use of (psychoactive) substances under the assumption of these substances being functional means in order to enhance his or her already proficient cognitive capacities (Wolff et al., 2014).

Depending on what type of substances are subsumed under the NE concept by the extant research on NE, the reported prevalence rates vary to a great extent. Lifestyle drug NE (e.g., Red Bull) is the most prevalent with reported rates as high as 89% (Mache et al., 2012). Prescription drug NE (e.g., Ritalin) and illicit drug NE (e.g., Speed) are reported at much lower rates of well below 10% (e.g., McCabe et al., 2005, 2012; Teter et al., 2006). However, the real prevalence rates for prescription drugs and illicit substances NE may be much higher as social desirability is likely to bias the results: Using randomized response techniques, the 1 year prevalence rate of prescription drug NE has for example been found to be as high as 20% (Dietz et al., 2013). In our further discussion of NE, we follow the behavioral definition of NE and subsume all three variants (i.e., lifestyle drug, or soft; Maier and Schaub, 2015, prescription drug and illicit drug NE) under the NE concept.

The potential negative effects of lifestyle NE substances on health are mostly unknown to the general public (Rath, 2012). For instance, high levels of caffeine and sugar in lifestyle products can be associated with nervousness, headaches, and tachycardia (Clauson et al., 2008). Even caffeine related deaths have been reported (Clauson et al., 2008).

The high prevalence rates (e.g., McCabe et al., 2005, 2012; Teter et al., 2006) and the potential negative health consequences (e.g., Clauson et al., 2008; Rath, 2012) that are associated with the most frequently used drugs underline the necessity to get a better understanding of *why* individuals start and/or *continue* to neuroenhance. However, past research on NE has been mostly conducted rather unsystematically, as for instance psychological correlates of NE behavior have been collected mostly in an explorative manner as part of epidemiological approaches at the expense of theory-driven, experimental approaches (e.g., Weyandt et al., 2009; Mache et al., 2012). As an exception, one recent study applied the *strength model of self-control* (Baumeister, 2003; Baumeister et al., 2007) to predict first time NE behavior in an experimental setting (Wolff et al., 2013). While not explicitly focused on NE behavior, another experiment has investigated the effect of Methylphenidate (a substance commonly used for NE) on self-control strength (Sripada et al., 2014). Finally, a very recent field study investigated the relationship of trait self-control strength and doping intentions (Chan et al., 2015). Based on the few theory-driven approaches to NE, self-control strength (or self-control demanding situations) seems to play an important role in NE behavior. We therefore think that the relationship between self-control and NE (and other forms of drug instrumentalization) warrants further investigation. We will explain our theoretical assumptions in more detail in the following sections.

## The Strength Model of Self-control

Self-control describes the ability to volitionally regulate ones’ behavior or predominant response tendencies in order to achieve a desirable goal (e.g., Baumeister et al., 1994, 2007). For instance, while being on a diet one has to resist tempting but high caloric drinks or snacks in order to achieve the long term goal of losing weight (e.g., Kahan et al., 2003). However, self-control does not always work and the strength model of self-control offers a potential explanation for lapses in self-regulatory behavior (Baumeister et al., 1998). According to Baumeister et al. (1994) all self-control acts (e.g., emotion regulation, persistence) are empowered by one global metaphorical resource. There are inter-individual differences in the capacity of this resource as some individuals are more adept in regulating themselves than others (i.e., trait self-control strength; e.g., Tangney et al., 2004). In general, this self-control strength has a limited capacity meaning that it can become temporarily depleted after having exerted self-control strength, which is a state labeled *ego depletion* (i.e., state self-control strength; e.g., Muraven and Baumeister, 2000). In a state of ego depletion self-control deficits are more likely to occur as there is less self-control strength available to volitionally regulate ones’ behavior (cf., Muraven and Baumeister, 2000). The effect of ego depletion is not domain-specific, meaning that previous acts of self-control in one domain (e.g., thought regulation) can have a negative carry-over effect on self-control performance in other, seemingly unrelated domains (e.g., emotion regulation; cf., Englert and Bertrams, 2013). Previous research has found a reliable effect of ego depletion on subsequent self-control performance as Hagger et al. (2010) report a medium-to-large effect of ego depletion on subsequent self-control in their meta-analysis.

Important for the present paper is the finding that under ego depletion individuals have a tendency to fall back onto their dominant behavioral tendencies (Govorun and Payne, 2006). For instance, restraint eaters are more likely to consume candy under ego depletion (Kahan et al., 2003; Hofmann et al., 2007) and in the same vein at-risk drinkers are more prone to relapses in a state of ego depletion (Ostafin et al., 2008).

## Self-control and NE

The strength model of self-control (e.g., Baumeister et al., 1994) allows for theoretically derived hypotheses regarding the self-control-NE relationship. Based on the strength models’ predictions and the existing empirical evidence (e.g., Hagger et al., 2010) this relationship does not seem to be a trivial one. First, NE and self-control seem to be associated in a reciprocal fashion: Self-control strength is associated with NE behavior (e.g., Wolff et al., 2013) and NE substances may also affect the availability of self-control resources (Sripada et al., 2014). Second, they seem to be associated both on a macro and on a micro level: On a macro level, trait differences in self-control are associated with differences in functional (e.g., doping in sports) and non-functional (e.g., illicit drugs) substance abuse (Chan et al., 2015). On a micro level, temporary depletion of self-control resources affects decisions to consume substances as a function of one’s history with such substance use behaviors (Wolff et al., 2013). In the following we will discuss these propositions in more detail.

### Self-control Resources Affect NE Behavior

As previously mentioned, individuals have a tendency to follow their regular habits or behavioral tendencies in a state of ego depletion (Govorun and Payne, 2006). Govorun and Payne found out that participants in a state of ego depletion were more likely to rely on their automatic behavioral tendencies which in that case was the tendency to rely on their stereotypes (i.e., an automatically activated response tendency) in a decision task. On the contrary, participants with temporarily available self-control strength were more likely to suppress their stereotypes and to respond in a more desirable manner.

Based on these findings, ego-depletion should thus differentially affect NE behavior as a function of one’s history with NE. If depleted, a regular user would be expected to neuroenhance as it is his or her dominant behavioral response tendency. This prediction is in line with the stereotypical image of an overwhelmed student who takes Ritalin^®^ to meet an assignment deadline or a manager who—before an important meeting—takes cocaine to perform better. However, for first-time NE users the predictions are reversed: If one has never used NE before, ego-depletion is predicted to elicit the dominant behavioral response which would then be to abstain from using a substance. This second prediction was investigated in a recent experiment (Wolff et al., 2013): Participants who had no history with NE were randomly assigned to a depletion or a non-depletion condition. After having worked on either a depleting or a non-depleting task, they were then informed that they would be asked to complete a cognitively demanding task after a short break. In this break they were given the opportunity to potentially enhance their performance with a caffeinated granulate. In line with the theoretical predictions, the depleted participants were actually significantly less likely to use the provided substance. So in this study, higher levels of state self-control strength were actually associated with a higher tendency to use NE to improve performance, indicating that higher levels of self-control strength were rather negative. This underlines the importance of self-control resources in the decision to neuroenhance for the first time and invites further research on the self-control-NE relationship. Most importantly, thus far the prediction that depletion leads to NE in habitual users has not been investigated and needs to be tested in future studies.

Chan et al. (2015) recently investigated how trait self-control strength is associated with athletes’ attitudes toward doping and the intentions of using substances to improve athletic performance. The authors found out that athletes with lower levels of trait self-control strength were more likely to have a heightened attitude and intention toward doping in general, and a reduced intention, behavioral adherence, and awareness of doping avoidance. Even though this study did not test how temporary levels of self-control strength affect actual NE behavior it gives a first indication that trait self-control strength also plays an important role in the self-control-NE relationship that needs to be investigated in more detail.

### NE Substances Can Affect Self-control Resources

In the previous section we discussed the complex relationship of ego depletion and the likelihood to use NE as a function of one’s NE experience. However, NE use may also be an adaptive behavior as it may help to replenish depleted self-control strength more quickly. A recent study investigated the effects of a popular NE substance on state self-control strength (Sripada et al., 2014). Specifically, the study revealed that Methylphenidate was effective in preventing ego-depletion states in an experimental setting. Participants from a Methylphenidate condition that had performed a primary self-control task did not display the typical impaired performance in a second self-control task, while participants from a control condition that did not consume Methylphenidate showed the typical ego depletion effect. Even though Sripada et al. (2014) did not explicitly focus on NE, their study gives an indication that some NE substances may alleviate ego depletion effects. This is important, as alleviation of depleted self-control strength might be a mediating variable in the subjective effectiveness individuals assign to an NE substance. This alleviation potential may thus be one explanation for the popularity of certain NE substances. Further, this research shows that self-control and NE seem to be associated in a bidirectional way. However, thus far it has not been sufficiently investigated how NE and ego depletion are interrelated. More research is needed to investigate how and why NE substances can replenish one’s self-control strength and how this potentially affects further NE behavior.

## Discussion

In the present paper, we argued for a theory-driven approach to investigate NE as thus far research in this field has been mostly conducted explorative. We identified the strength model of self-control (Baumeister et al., 1998) as a promising candidate theory. Self-control and NE seem to be interrelated in a bidirectional manner: Self-control resources affect the initiation of NE behavior depending on one’s personal NE experience (Wolff et al., 2013). Trait self-control strength is also related with one’s attitude toward NE and the intentions of consuming NE (Chan et al., 2015). NE substances can also affect the availability of self-control resources as certain substances may lead to a quicker revitalization of depleted self-control strength (Sripada et al., 2014). We reviewed research that can be seen as a first step to investigate both directions and outlined further research questions that would allow for theory-driven experimental research on NE.

The ethical verdict and policy implications on NE are still heavily debated (e.g., Farah et al., 2004; Greely et al., 2008; Forlini and Racine, 2009). The goal of this article was not to take a side in this debate as we do not recommend taking certain substances to replenish depleted self-control strength. We rather wanted to provide a theoretical backdrop for conducting psychological research on the initiation and the effects of NE. We are convinced that the complex relationship of NE and self-control warrants further investigation and will allow for a deeper understanding of this behavioral trend.

## Author Contributions

CE and WW substantially contributed to the writing of the manuscript. Both authors approve the final version of the manuscript. The authors agree to be accountable for all aspects of the work in ensuring that questions related to the accuracy or integrity of any part of the work are appropriately investigated and resolved.

### Conflict of Interest Statement

The authors declare that the research was conducted in the absence of any commercial or financial relationships that could be construed as a potential conflict of interest.
